# Left suboccipital supracerebellar transtentorial approach for resection of tectal cavernous malformation

**DOI:** 10.3171/2019.10.FocusVid.19394

**Published:** 2019-10-01

**Authors:** Hussam Abou-Al-Shaar, Timothy G. White, Ivo Peto, Amir R. Dehdashti

**Affiliations:** 1 Department of Neurological Surgery, University of Pittsburgh Medical Center, Pittsburgh, Pennsylvania; and; 2 Department of Neurosurgery, Hofstra Northwell School of Medicine, Manhasset, New York

**Keywords:** brainstem, cavernous malformation, transtentorial approach, safe entry zone, lateral mesencephalic sulcus, video

## Abstract

A 64-year-old man with a midbrain cavernoma and prior bleeding presented with a 1-week history of diplopia, partial left oculomotor nerve palsy, and worsening dysmetria and right-sided weakness. MRI revealed a hemorrhagic left tectal plate and midbrain cavernoma. A left suboccipital supracerebellar transtentorial approach in the sitting position was performed for resection of his lesion utilizing the lateral mesencephalic sulcus safe entry zone. Postoperatively, he developed a partial right oculomotor nerve palsy; imaging depicted complete resection of the cavernoma. He recovered from the right third nerve palsy, weakness, and dysmetria, with significant improvement of his partial left third nerve palsy.

The video can be found here: https://youtu.be/ofj8zFWNUGU.

## Transcript

Here we present the surgical treatment of a symptomatic tectal cavernous malformation in a 64-year-old man who is known for this lesion for several years. However, he presented recently with an episode of bleeding resulting in dysmetria and right-sided weakness. He also presented about a week before transfer to our center with a new left-sided third nerve palsy, which is quite significant, almost a complete palsy. Imaging revealed a new hemorrhage in the brainstem cavernous malformation in the tectal region, as you can see, in this axial T2 imaging. The picture shows that the cavernous malformation extends to the midline of the brainstem and has a quite of a superior extension to the top of the tectum. The coronal pictures showing the characteristics of this cavernous malformation with its superior extension, which is also important in terms of decision-making for surgical approach, as you can see in this particular picture here. The sagittal shows that a supracerebellar approach with the addition of transtentorial exposure can give a direct exposure of the posterior aspect of the cavernous malformation.

We decided to use the lateral mesencephalic sulci as a safe entry zone to this lesion, and the indication is obviously based on episodes of rehemorrhage and patient’s current symptoms of cranial nerve palsy, right-sided dysmetria, and weakness. We decided to perform the surgery in the sitting position, which will allow the cerebellum to fall with gravity. A slight paramedian left-sided incision. The patient’s feet are elevated at the level of the right atrium to decrease the chance of gas emboli.

A craniotomy was performed in the suboccipital region, exposing almost to the foramen magnum on the left side. A small opening of the dura at the foramen magnum allows release of CSF and that will facilitate opening of the dura superiorly in a C-like fashion, and that will give an unobstructed view in the supracerebellar region towards the tentorial incisura. You can see the branch of the superior cerebellar artery, the trochlear nerve, dissection of the arachnoid, and here the lateral mesencephalic vein, and we use the navigation to confirm the location of the cavernous malformation and use the lateral mesencephalic safe entry zone, which is right here, to get to the cavernous malformation itself. Once identification of the cavernous malformation is confirmed, a piecemeal resection is being performed. Obviously, here an en bloc resection is impossible due to the size of this lesion. After resection of the lesion centrally and debulking, we will find, as you can see here, the interface between the cavernous malformation and the brainstem tissue. There are some venous pockets that are part of the cavernous malformation that have to be resected. Obviously, if we find a developmental venous anomaly we will preserve, but in this particular case, I did not clearly identify that; however, the lesion is quite distinguishable from the normal brain parenchyma. Here, we resect the lateral aspect, the lateral mesencephalic vein is being pushed laterally, and we pull the lesion into the surgical exploration view from lateral to medial, and also once its debulked we are also able to bring the lesion from superior down. As you can see, we can pull the lesion, but still some abnormal part of the lesion that is stuck to the brainstem parenchyma or to the hemosiderin that is being resected in a microsurgical fashion. Once we are done with this part when the core of lesion is removed, we would like to make sure that there is no remnant on the superior aspect and therefore that is then removed with opening of the tentorium. Here, that is the superior part. We pull the lesion down, there is some bleeding from the cavernous malformation that is controlled with coagulation. In order to make sure that there is no remnant left superiorly, despite pulling the lesion down, we add this transtentorial exposure, we see the wall of the third ventricle, and final look into the surgical cavity, confirming absence of any remnant of the malformation.

A routine closure is performed. This patient woke up with a new deficit, which was a right-sided third nerve palsy of about 60%–70%, and that fortunately improved to complete normal function at 6 months. Even the left third nerve palsy improved quite a bit. Postop MRI showed complete resection of the lesion; there is remnant of hemosiderin in the superior anterior aspect, but otherwise the patient has a good recovery.

Thank you for your attention.

## Time points

1:56 MRI depicting lateral mesencephalic sulcus safe entry zone
2:01 Sitting position with feet elevated at the level of right atrium for a left suboccipital supracerebellar transtentorial approach
2:27 Opening the dura in C-shaped fashion
2:42 Exposure of the superior cerebellar artery, trochlear nerve, arachnoid, and lateral mesencephalic vein to reach the tectal cavernous malformation
3:10 Piecemeal resection of the cavernous malformation
4:40 Cutting the tentorium to expose the superior aspect of the cavernous malformation
